# Randomised control trial of virtual reality in cognitive rehabilitation: effectiveness and near-transfer effect for stroke patients

**DOI:** 10.1186/s40359-025-03135-8

**Published:** 2025-07-18

**Authors:** Jovita Janavičiūtė-Pužauskė, Raimonda Petrolienė, Loreta Zajančkauskaitė-Staskevičienė, Andrius Paulauskas, Liuda Šinkariova

**Affiliations:** 1 Department of Psychology, Vytautas Magnus University, Jonavos g. 66, Kaunas, 44138 Lithuania; 2 Department of Health Psychology, Lithuanian University of Health Sciences, Tilžės g. 18, Kaunas, 47181 Lithuania

**Keywords:** Cognitive rehabilitation, Stroke, Egocentric perspective, Allocentric perspective, Near transfer effect

## Abstract

**Background:**

This study aims to determine whether cognitive training based on an egocentric perspective leads to greater improvements in target functions compared to training using an allocentric perspective or conventional rehabilitation. Additionally, it seeks to examine whether egocentric perspective-based training enhances the near transfer effect, resulting in improved other cognitive functions compared to allocentric perspective-based training and conventional rehabilitation.

**Methods:**

132 stroke patients (65.9% males) aged 31 to 83 (mean age 62.54 ± 10.62) undergoing inpatient rehabilitation were randomly assigned to one of the three groups: allocentric (*N* = 40) or egocentric perspective-based (*N* = 46) selective visual attention and short-term visual memory training and control (conventional rehabilitation) group (*N* = 46). Evaluations were performed before (at the beginning of rehabilitation for control group) and after the intervention (two weeks after pre-test for control group). The target functions included the changes in short-term visual memory, visual-spatial attention, attention-orientation, while near transfer effect included the changes in visuo-construction abilities, task switching, verbal memory, verbal fluency, language, visuo-spatial abilities, general cognitive function. To investigate how different perspectives affect selective visual attention and short-term visual memory training and near-transfer effect over time, while accounting for baseline cognitive scores, were conducted mixed repeated measures ANOVA and ANCOVA (controlled for age, years of education, lesion location, and days since stroke). A priori power analysis and effect sizes using Cohen’s d_z_ for matched pairs were calculated.

**Results:**

The results showed that cognitive rehabilitation tasks based on egocentric perspective in combination to conventional rehabilitation are effective in improving attention-orientation and cause near transfer effect on spatial abilities and general cognitive functioning. While tasks based on allocentric perspective in combination to conventional rehabilitation are effective in improving short-term visual memory and cause near transfer effect on spatial abilities.

**Conclusions:**

The cognitive improvements and near-transfer effects observed in our results indicate that combining conventional rehabilitation with tasks based on egocentric and allocentric perspectives integrated in virtual reality environments can enhance rehabilitation effectiveness. However, further studies are required to confirm the generalizability of these findings.

**Trial registration:**

The present study, along with its protocol, was retrospectively registered in the ISRCTN clinical trial registry on January 3, 2024 (10.1186/ISRCTN14922230).

**Supplementary Information:**

The online version contains supplementary material available at 10.1186/s40359-025-03135-8.

## Background

One of the primary objective of rehabilitating stroke survivors is to enhance their cognitive function, especially selective visual attention and short-term visual memory, through comprehensive and optimal interventions. These functions are fundamental cognitive processes that are closely linked to other cognitive domains. Moreover, impairments in selective visual attention and short-term visual memory are very common among individuals who have experienced a stroke. Even after rehabilitation, deficits in attentional functions often persist or may even worsen [1, 2].

Cognitive neurorehabilitation is based on two paradigms– functional compensation and functional restoration. In order to minimize the consequences of cognitive impairment, based on a compensatory strategy patient learn strategies that help to functioning in everyday life [3]. While restoration paradigm seeks to restore the cognitive functions through neuroplasticity and patients train their impaired functions. The compensation paradigm is typically more effective for patients with severe impairments where the restoration of function is not feasible [4]. In contrast, the restoration paradigm leverages neuroplasticity, particularly in the early months following a stroke, to promote recovery of cognitive functions. Evidence suggests that rehabilitation tasks designed within the restorative framework leads to more significant and faster improvements in cognitive abilities for post-stroke patients [5, 6]. Deficits in attention are often addressed through restorative computer-based tasks, as these methods can effectively employ rapidly and repetitively presented stimuli [7]. It is crucial to select rehabilitation methods that enhance overall cognitive functioning by targeting fundamental cognitive domains, such as selective visual attention and short-term visual memory, to optimize the near-transfer effect that is based on the overlapping neural networks and transfer of learning [8, 9].

The near transfer effect is based on *The Principle of Identical Elements theory*. It states that the training effect gained from practicing one function can be applied to improve another related function, and this transfer of learning occurs under similar conditions that means transfer of learning occurs when both functions are performed in similar situations or environments [10]. Furthermore, Barnett & Ceci (2002) classify near transfer as occurring when there is substantial overlap between the trained and target functions. This suggests that when fundamental cognitive functions– such as attention or memory, which are interconnected with other cognitive domains– are trained, improvements are not limited to the targeted function but can also extend to related cognitive abilities due to their shared elements and processing demands. According to Von Bastian and Oberauer [11], the findings regarding transfer effects following cognitive training remain controversial. One contributing factor to this controversy is the variability in training characteristics across studies, along with the lack of a comprehensive description of the underlying rationale for the training. This study will focus on the effects of training task characteristics such as allocentric and egocentric perspectives and their influence on the effectiveness of cognitive rehabilitation.

In this study, selective visual attention and short-term visual memory are trained, with allocentric and egocentric perspectives as key session characteristics. Allocentric and egocentric perspectives refers to the conceptual level, whereas the phenomenological level corresponds to third-person and first-person perspectives. The allocentric perspective refers to an environment-centered way of processing visual information, where objects are understood in relation to other objects or landmarks, this is known as object-to-object processing. In contrast, the egocentric perspective is self-centered, processing visual information based on the position of objects relative to oneself [12]. These different perspectives and their significance in various contexts are challenging to study in isolation. Therefore, non-immersive and immersive virtual reality methods respectively are used to convey allocentric and egocentric perspectives [13].

Allocentric and egocentric perspectives may influence the effectiveness of target functions and the near transfer effect differently and can be beneficial in the combination of conventional rehabilitation. Both perspectives are associated with visual information processing [12], and while these factors have been examined in the context of assessing navigational abilities and spatial memory, their role in attention and memory training in the context of rehabilitation remains unexplored. The observed greater stability of egocentric spatial processing across the human lifespan and its diminished vulnerability to neurological pathologies, as compared to allocentric spatial processing [14, 15]. Egocentric perspective-based tasks are particularly effective at producing near transfer effects– improvements on tasks that are similar to the training activity– due to their high level of participant involvement, multisensory stimulation and optimization of neuroplasticity [16, 17]. This supports the hypothesis that cognitive training interventions designed from an egocentric perspective may confer enhanced therapeutic efficiency. Drawing upon the existing literature, the present study is aimed at addressing two specific research gaps. Firstly, we aim to explore whether cognitive training based on an egocentric perspective yields a greater improvement in target functions compared to tasks based on an allocentric perspective or conventional rehabilitation. Secondly, we aim to investigate whether cognitive training based on an egocentric perspective brings about a greater near transfer effect that results in improved other cognitive functions (visuo-construction abilities, task switching, verbal memory, verbal fluency, language, visuo-spatial abilities, general cognitive functions) compared to training based on an allocentric perspective or conventional rehabilitation. This will lead to a better understanding of stroke patients’ rehabilitation progress and optimization.

## Methods

A randomized controlled trial with a parallel design was conducted in Neurological unit at Abromiškės Rehabilitation Hospital. This study was conducted in accordance with the ethical principles outlined in the Declaration of Helsinki. All procedures were approved by the Vilnius Regional Biomedical Research Ethics Committee (No. 2022/2-1408-880). Written informed consent was obtained from all participants prior to their inclusion in the study. The present study, along with the study protocol, was retrospectively registered in the ISRCTN clinical trial registry (10.1186/ISRCTN14922230).

Patients who had suffered a stroke were invited to participate in the study. Participants were recruited between February 15, 2022, and October 1, 2024. The following inclusion criteria were applied to all participants: (1) to have the confirmed diagnosis of stroke, (2) no severe cognitive impairment detected using Addenbrooke’s Cognitive Examination III cut-off score of 38 [18], (3) the native language is Lithuanian, (4) arrived at the rehabilitation hospital at least 3 days ago, (5) being able to sit for 30 min. The following exclusion criteria were applied to all participants: (1) to be over the age of 85 years, (2) to have epilepsy or psychiatric diagnoses established, (3) to have a severe degree of aphasia which would have been diagnosed by a speech therapist and characterized by difficulty in maintaining a coherent conversation or understanding spoken language, (4) to experience unilateral neglect which would have diagnosed by neurologist, (5) to be characterized by severe impairment of both hands, (6) to have other communication impairments that may prevent the patient from understanding task instructions or the purpose of the study.

Participants were allocated to one of the three groups using a simple randomization method, where assignment was determined by the roll of a dice. To avoid researcher bias, randomization was carried out before the pre-assessment, but a double-blind allocation design was not implemented due to lack of human resources. The allocation of participants to groups was conducted by a single researcher (JJP), while assessments were performed independently by different researchers (RP and LZS). The day following the pre-assessment, participants assigned to the Allocentric perspective (AP) or Egocentric perspective (EP) groups, in addition to conventional rehabilitation, began 30-minute training sessions focused on selective visual attention and short-term visual memory (5 days per week for two weeks) respectively based on allocentric or egocentric perspective. Meanwhile, participants in the control group (C) continued with conventional rehabilitation. Two weeks after the pre-assessment, a post-assessment was conducted to evaluate outcomes.

### Measures

Sociodemographic and clinical information was collected during the pre-assessment. The collected sociodemographic data included gender, age, years of education, place of residence, and dominant hand preference. Clinical information covered stroke type, stroke localization, and stroke stage. The interventions used in this study specifically target the training of selective visual attention and short-term visual memory; therefore, these functions, along with attention-orientation, will be considered the target functions. In contrast, functions associated with the near-transfer effect include verbal fluency, verbal memory, language, spatial ability, general cognitive functioning, task switching, and working memory.

### Measurements of target functions

*Short-term visual memory* was assessed using the Medical College of Georgia Complex Figures (MCGCF) Forms A and B [19], which are based on the Rey-Osterrieth complex figure test. The test consists of two parts: copying and recalling after 3 min. Each part was scored and timed. The maximum possible score is 36.

*Selective visual attention* was measured using the Trail Making Test Form A (TMT-A) [20]. *Attention-orientation* was measured via the Addenbrooke’s Cognitive Examination III (ACE-III) Attention-orientation subscale [21].

### Measurements of near transfer effect

General cognitive functions were assessed using the Addenbrooke’s Cognitive Examination III (ACE-III) Forms A and B [21]. The instrument measures five domains: attention-orientation (time and location orientation, attention sustainability), verbal memory (remember and recall three words, address, semantic memory task), verbal fluency (phonemic and semantic fluency tasks), language (writing, reading, naming tasks) and spatial ability (copy, clock drawing, incomplete letter naming task). The maximum possible score for the whole test is 100.

Task switching was assessed using the Trail Making Test Form B (TMT-B) [20]. The instrument assesses attention and visual search, with a score of Part B for task shifting and working memory [22]. The time in seconds and the number of errors were recorded for each part. To eliminate the effect of psychomotor speed, an additional B/A score was computed and used to assess executive functions [22].

### Intervention

To test the hypotheses suggested in the present study, selective visual attention and short-term visual memory training tasks [23] were prepared according to two different task features: allocentric and egocentric perspectives.

The tasks were developed in four phases [24]: (1) the initial development phase, (2) the program construction, (3) the program testing, (4) the final development phase. Tasks were created based on principles of Flow theory [25] and principles of neurorehabilitation tasks [26], which overlap. It is important to mention that these principles also fit to restorative paradigm [7]. Similar studies focusing on memory and attention training, alongside other cognitive functions, often involve scenarios where participants must remember shopping lists or navigate to a shopping centre and etc [27, 28]. In order to adapt the tasks for stroke patients, we determined that the scenarios should be stationary, meaning no movement within the virtual environment, to prevent cybersickness [29]. The task scenarios are as follows: (1) recall the sequence of presented food products, and (2) recall food products from memory. Both scenarios refer to selective visual attention and short-term visual memory training. The task scenarios will be described in more detail below.

Both tasks contain the same kitchen environment. The participant appears in front of the cupboards where the food products appear. The same appearance of food products used in the tasks can be found in every Lithuanian supermarket. Audible nonverbal feedback was provided to participants in both tasks: upon placing the product and upon completion of the task. Additionally, during the task participants were allowed to make up to three errors. From the first to fifth sessions the participants worked on the first task and from the sixth to tenth sessions the participants worked on the second task [23]. The highest achieved number of remembered objects in each session was recorded.

**Fig. 1:**
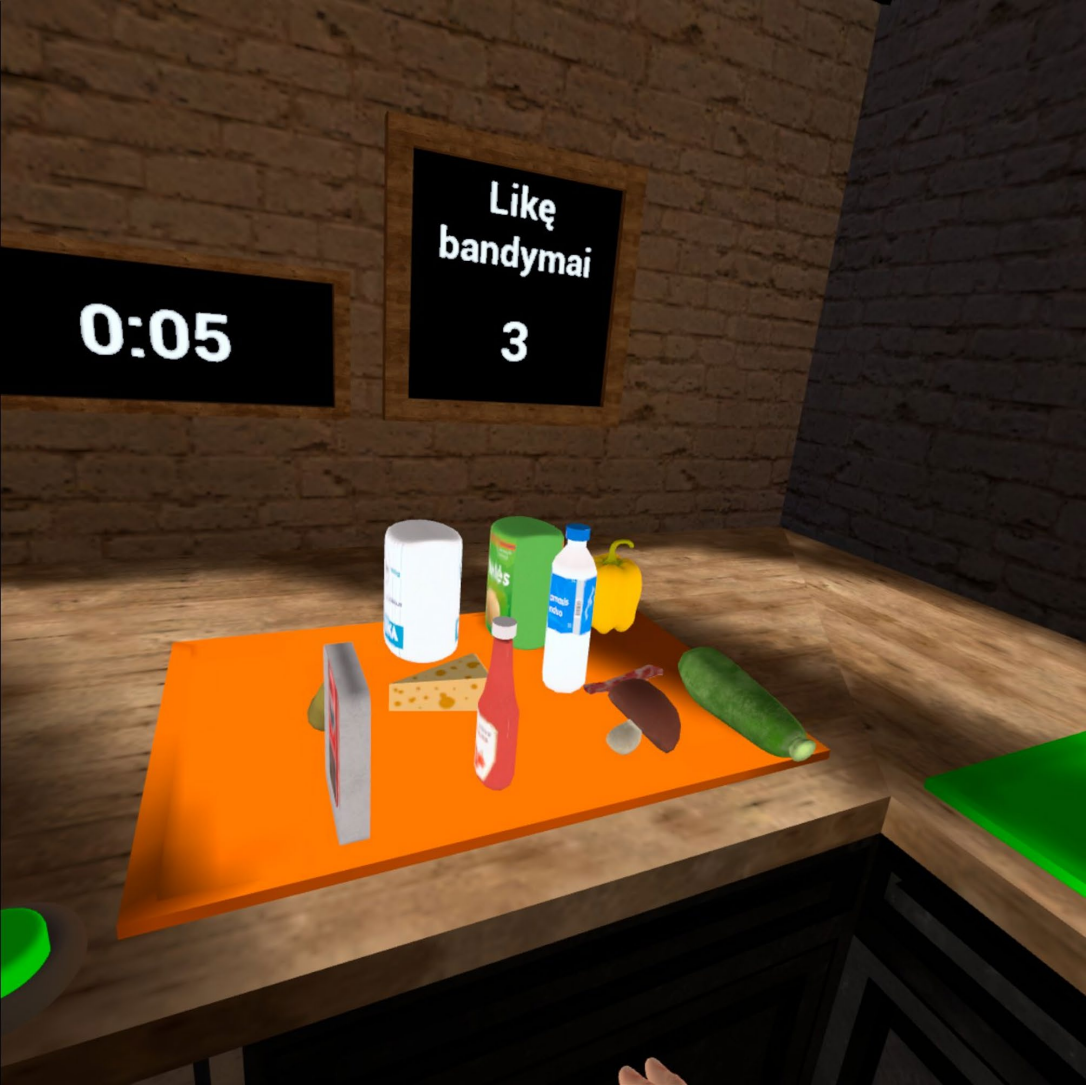
The illustration of the first task based on egocentric perspective

During the first task, the participant was instructed to memorize the food products (3 s per product is given to memorize) that appeared on the green tray on the right side (Fig. 1). After that, on the orange tray, more products appear, and participant was asked to recall previous products (the order of recalling was not important) and put those products on the green tray. Participants do not need to remember the exact location of the product. This task starts with one food product and after two successful errorless times, tasks’ difficulty increases by one product.

During the second task, the participant was instructed to memorize the sequence (products highlighted in a random order). The participant sees food products on the cupboard and three (3 s per product is given to memorize) of all products highlights in random order. Participant was asked to pick up the products in the same order and put them into the bowl. This task starts with the sequence of three food products and after two successful errorless times, tasks’ difficulty increases by one product.

Tasks based on allocentric perspective which provided third-person perspective experience were incorporated into the non-immersive virtual reality (Samsung Galaxy Tab S8 Plus). Meanwhile, tasks based on egocentric perspective which provided first-person perspective experience were incorporated into the immersive virtual reality (Oculus Quest 2 head-mounted display).

In addition, to assess the suitability and construct validity of the intervention, expert evaluations, suitability testing, and a manipulation check were conducted. Experts reviewed the procedure and tasks, concluding that they are appropriate for training selective visual attention and short-term visual memory in stroke patients, with a high content validity index (S-CVI/M ≥ 0.97). Furthermore, the tasks were tested in a separate study group using the System Usability Scale [30]. The results indicated that both allocentric perspective-based tasks (M = 80.41) and egocentric perspective-based tasks (M = 81.52) are suitable for stroke patients and rated as easy to use. Manipulation check was completed by asking participants in two experimental groups about the experienced perspective. The majority of participants (91,9%) in allocentric perspective group responded that they experienced third-person perspective, as well as the majority of egocentric perspective group participants (90.9%) responded that they experienced first-person perspective. When 80% threshold is reached construct validity is sufficient [31]. Questions about adverse symptoms (e.g., dizziness, vertigo, breathlessness, impaired vision, or coordination issues) during the intervention were included to assess feasibility. However, participants did not report experiencing any adverse symptoms during the study.

### Participants

**Fig. 2:**
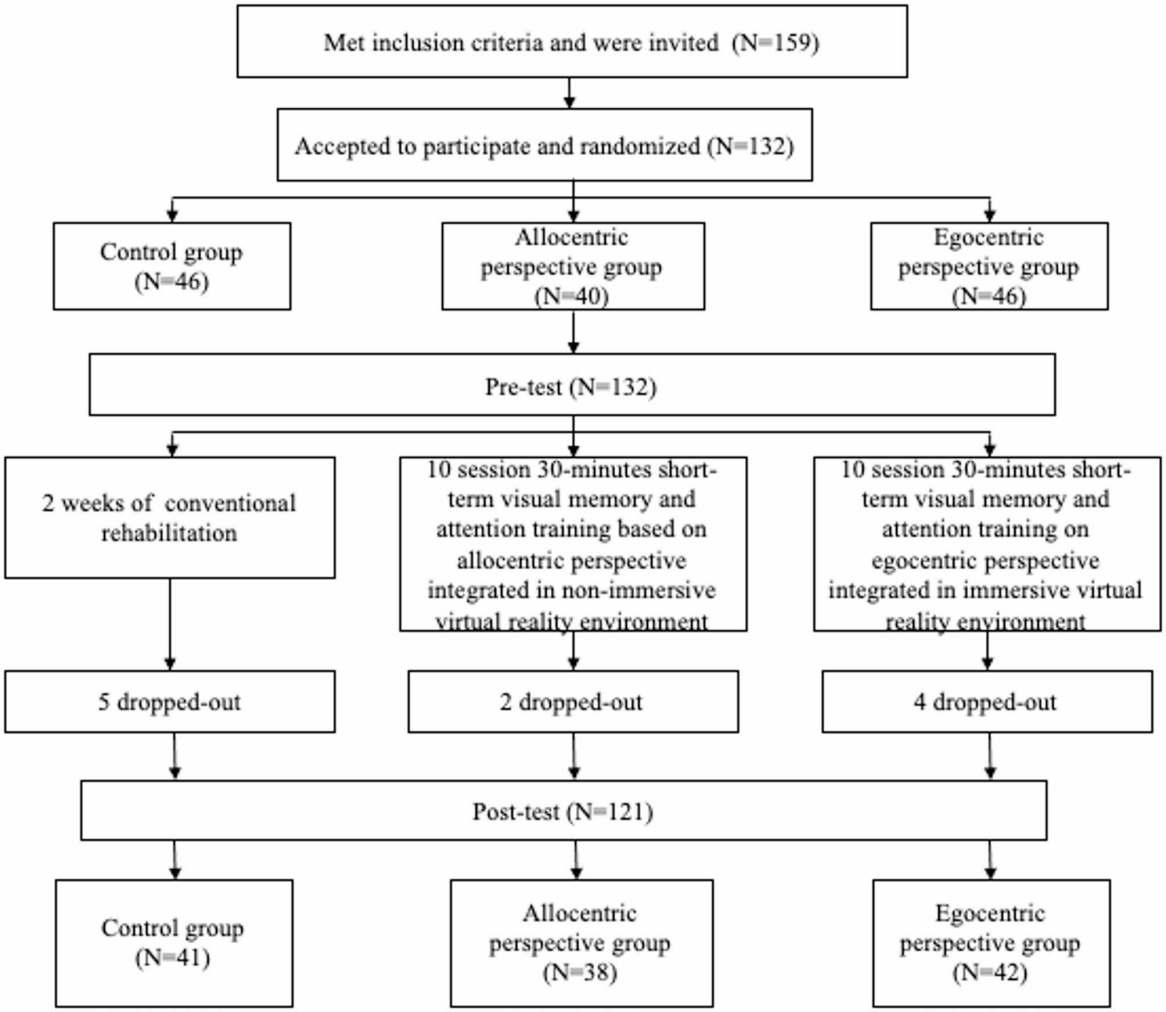
The diagram of the study design

A total of 132 patients, aged 31 to 83 (mean age 62.54 ± 10.62), undergoing inpatient rehabilitation agreed to participate in the study. More than half (65.9%) of participants were males. During the study, eleven participants withdrew from the study (see Fig. 2), and their data were excluded from the subsequent analysis. Seven of them did not complete the post-test due to early discharge from the rehabilitation hospital, while four participants discontinued participation because of a lack of motivation. Excluding patients who withdrew after the pre-test, 121 stroke patients remained in the post-test.

Demographic characteristics and clinical data of 121 participants are shown in Table 1 while pre-test scores across groups are shown in Table 2.

**Table 1:** Demographic, clinical and functional data of participants in all three groups during the pre-test

		Control group (*N* = 41)	AP group (*N* = 38)	EP group (*N* = 42)	Total (*N* = 121)	Statistical criteria, *p*
Age (mean ± SD)		64.07 ± 8.27	62.37 ± 13.08	60.43 ± 9.8	62.27 ± 10.52	F(2,118) = 1.252, *p* =.290
Sex (%)	Males	31 (75.6)	20 (52.6)	28 (66.7)	79 (65.3)	χ^2^(2) = 4.649, *p* =.098
	Females	10 (24.4)	18 (47.4)	14 (33.3)	42 (34.7)	
Dominant hand (%)	Right-handed	41 (100)	34 (89.5)	40 (95.2)	115 (95)	χ^2^(4) = 4.642, *p* =.326
	Left-handed	0	2 (5.3)	1 (2.4)	3 (2.5)	
	Ambidextrous	0	2 (5.3)	1 (2.4)	3 (2.5)	
Residence (%)	City	25 (61)	26 (68.4)	25 (59.5)	76 (62.8)	χ^2^(2) = 0.765, *p* =.682
	Village	16 (39)	12 (31.6)	17 (40.5)	45 (37.2)	
Years of education (mean ± SD)		13.27 ± 1.63	13.95 ± 1.58	13.38 ± 1.86	13.52 ± 1.71	F(2, 118) = 1.797, *p* =.170
Marital status (%)	In the romantic relationships	31 (75.6)	21 (55.3)	28 (66.7)	80 (66.1)	χ^2^(2) = 3.653, *p* =.161
	Not in the romantic relationships	10 (24.4)	17 (44.7)	14 (32.6)	41 (33.9)	
**Clinical data**						
Lesion side (%)	Right sided	23 (56.1)	17 (45.9)	21 (50)	61 (50.8)	χ^2^(4) = 5.267; *p* =.261
	Left sided	16 (39)	20 (54.1)	21 (50)	57 (47.5)	
	Not identified	2 (4.9)	0	0	2 (1.7)	
Stroke type (%)	Ischemic	33 (80.5)	32 (84.2)	38 (90.5)	103 (85.1)	χ^2^(2) = 1.671; *p* =.434
	Haemorrhage	8 (19.5)	6 (15.8)	4 (9.5)	18 (14.8)	
Days after stroke		346.51 ± 773.96	246.42 ± 843.11	720.83 ± 2097.21	442.71 ± 1393.89	F(2, 117) = 1.672, *p* =.277
Number of strokes (%)	One	35 (85.4)	29 (76.3)	40 (95.2)	104 (86)	χ^2^(2) = 5.933; *p* =.051
	More than one	6 (14.6)	9 (23.7)	2 (4.8)	17 (14)	

C = control group; AP = allocentric group; EP = egocentric group

The analysis revealed that there were no statistically significant differences in demographic characteristics and clinical data between the groups (Table 1). Also, the groups were homogenous based on the pre-test scores of cognitive functions (Table 2).

**Table 2:** Cognitive function scores across the three groups during the pre-test

	EP (*N* = 42)		C (*N* = 41)		AP (*N* = 38)		F (df. error)	η^2^
	M	SD	M	SD	M	SD		
ACE-III Attention-orientation	15.85	2.22	16.07	2.19	16.68	1.54	(2.128) 1.882	0.029
ACE-III Verbal Memory	19	4.04	18.67	5.03	19.78	4.03	(2.128) 0.675	0.01
ACE-III Verbal fluency	8.37	3.2	7.91	2.67	8.6	2.94	(2.128) 0.609	0.009
ACE-III Language	23.15	2.84	23	3.4	23.7	2.59	(2.128) 0.638	0.01
ACE-III Spatial abilities	14.28	2.21	13.73	2.06	14	1.68	(2.128) 0.851	0.013
ACE-III total	80.57	10.64	79.4	11.16	82.75	9.01	(2.128) 1.130	0.017
TMT A	58.87	34.59	65.09	32.21	56.3	29.75	(2.128) 0.843	0.013
TMT B	147.4	83.04	164.7	79.08	152.65	73.67	(2.128) 0.56	0.009
TMT B/A	2.83	1.38	2.73	1.21	2.95	1.32	(2.126) 0.29	0.005
MCGCF copy score	32.75	4.82	33.52	3.19	34.7	2.03	(2.115 3.069	0.05
MCGCF copy time	157.59	83.66	157.22	64.01	137	58.61	(2.115 1.151	0.02
MCGCF 3-min recall score	16.96	10.45	15.18	7.95	16.06	7.13	(2.116 0.423	0.007
MCGCF 3-min recall time	107.05	51.85	112.12	49.08	107.58	45.9	(2.116 0.130	0.002

Statistical significance = *p < 0,05, **p < 0,01, ***p < 0,001; MCGCF = Medical College of Georgia Complex Figures Forms A and B; TMT = Trail Making Test Forms A and B; ACE-III = Addenbrooke’s Cognitive Examination III; C = control group; AP = allocentric group; EP = egocentric group

To control the influence of confounding variables on the results, information about other procedures that participants underwent during rehabilitation was collected. Those procedures were grouped as facilities of motor recovery (e.g. exercises in the pool, exercises in the gym), occupational therapy, physiotherapy (e.g. laser, electrostimulation, microwave therapy). Groups were identical in terms of the number of motor recovery procedures F(2,118) = 0.930, *p* =.397, occupational therapy F(2,118) = 0.851, *p* =.430, physiotherapy F(2,117) = 0.807, *p* =.448, speech therapy F(2,118) = 0.250, *p* =.779, all procedures F(2,117) = 0.272, *p* =.762.

### Statistical analysis

In our study, statistical analyses were conducted using IBM^®^ SPSS^®^ Statistics 22.0. Descriptive statistics were computed for all variables. To investigate how different perspectives affect selective visual attention and short-term visual memory training and near-transfer effect over time, while accounting for baseline cognitive scores, were conducted mixed repeated measures ANOVA and ANCOVA. The analysis considered cognitive improvement as the dependent variable, treatment type as the independent variable, and age, years of education, lesion location, and days since stroke as covariates. Additionally, a priori power analysis and effect sizes using Cohen’s d_z_ for matched pairs were calculated via G*Power 3.1 [32]. These effect sizes allowed for the comparison of groups by quantifying the magnitude of the change in each group, providing a standardized measure of effect independent of sample size [33]. The effect size was interpreted as small (0.1), medium (0.3) and large (> 0.5) [34]. Based on a priori power analysis a sample size of 128 stroke patients is required to maintain 80% power and detect small effect sizes (0.25) in a randomized controlled trial with pre- and post-tests.

## Results

### Effectiveness on target functions

Results of pre-test and post-test changes within each group revealed that there was no significant improvement in the control group. Meanwhile, participants in the allocentric perspective group improved their short-term visual memory and selective visual attention. Participants in the egocentric perspective group significantly improved all the target functions (short-term visual memory, attention-orientation, selective visual attention). For more detailed results about changes within group and effect sizes see Table S1 in Supplementary material.

**Fig. 3:**
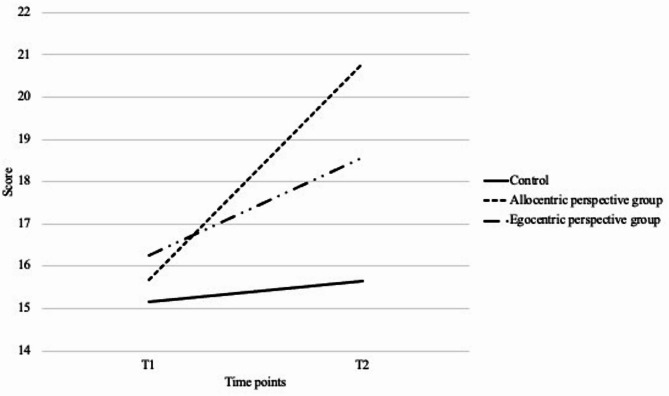
Changes of short-term visual memory score across three groups

Significant interaction effects were found for two of three target functions. Additionally, a significant interaction effect between time and group was found (F (2, 108) = 5.3, *p* <.01, η^2^ = 0.089) on short-term visual memory. Post hoc comparisons using Bonferroni correction indicated that the allocentric perspective group (M_1_ = 15.67, SD_1_ = 7.1; M_2_ = 20.79, SD_2_ = 8.26) showed greater improvements (*p* =.033) compared to the control group (M_1_ = 15.17, SD_1_ = 8.08; M_2_ = 15.64, SD_2_ = 8.34). The interaction remained significant after including the covariates, F (2, 100) = 3.898, *p* <.05, η^2^ = 0.072 (Fig. 3).

**Fig. 4:**
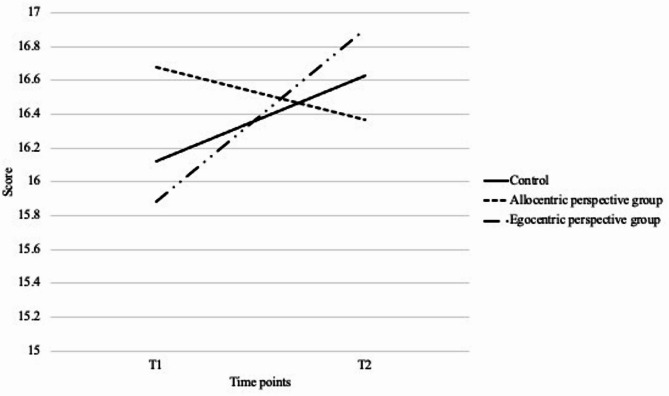
Changes of attention-orientation score across three groups

Examining *attention-orientation* improvement there was a significant interaction effect between time and group (F (2, 118) = 7.333, *p* <.001 η^2^ = 0.111). Post hoc comparisons using Bonferroni correction indicated that the egocentric perspective group (M_1_ = 15.88, SD_1_ = 2.27; M_2_ = 16.9, SD_2_ = 1.72) showed greater improvements (*p* =.001) compared to the allocentric perspective group (M_1_ = 16.68, SD_1_ = 1.58; M_2_ = 16.37, SD_2_ = 2.05). The interaction remained significant after including the covariates, F (2, 110) = 6.666, *p* <.01, η^2^ = 0.108 (Fig. 4).

**Fig. 5:**
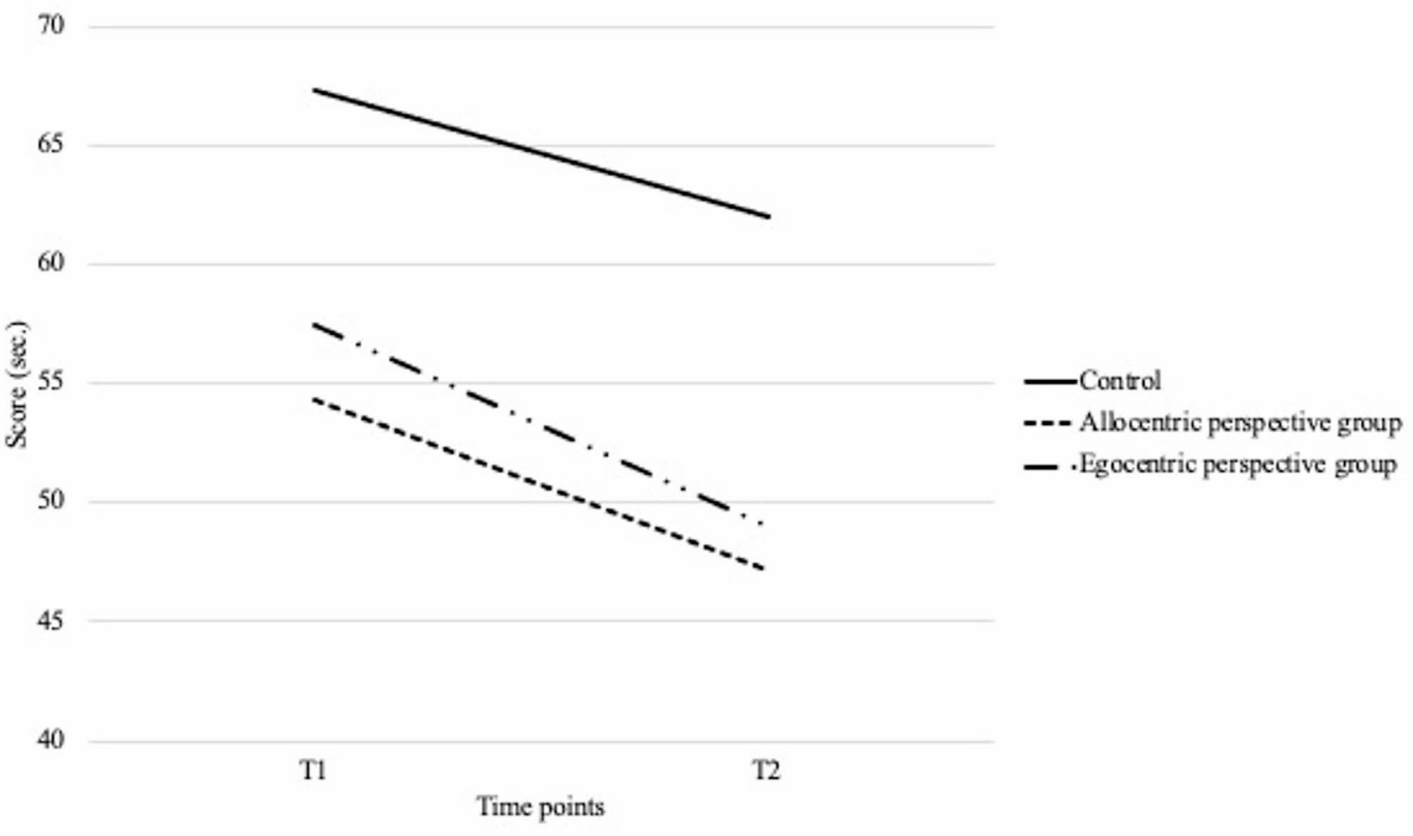
Changes of selective visual attention score across three groups

However, examining selective visual attention improvement the interaction effect between time and group was not significant (F (2, 118) = 0.306, *p* >.05, η² = 0.005). This non-significance persisted after including the covariates, F (2, 110) = 0.691, *p* >.05, η² = 0.012, suggesting that the groups showed similar improvements over time (Fig. 5).

### Effectiveness on near-transfer effect

Comparison of the results of the primary and secondary assessments within each group showed that the control group did not show any difference in the indicators of directly untrained functions. Meanwhile, the participants in the allocentric perspective group improved spatial abilities, while egocentric perspective group improved task switching, language, spatial abilities, verbal fluency and general cognitive functioning. For more detailed results about changes of near-transfer effect measures within group and effect sizes see Table S2 in Supplementary material.

Significant interaction effects between group and time comparing three groups were found for two near-transfer measures. An interaction effect between time and group on visuospatial abilities was not significant, F(2, 108) = 0.662, *p* >.05, η^2^ = 012. This non-significance persisted after including the covariates, F(2, 110) = 0.912, *p* >.05, η² = 0.018, suggesting that the groups showed similar visuospatial improvements over time. However, interaction effect between time and group on spatial abilities was significant without covariates F(2, 118) = 3.721, *p* <.05, η^2^ = 059, and including covariates F(2, 110) = 4.237, *p* <.05, η^2^ = 0.072. This interaction revealed that improvement of spatial abilities were greater in allocentric perspective and egocentric perspective groups comparing to control group.

Additionally, an interaction effect between time and group on task switching (F(2, 115) = 0.156, *p* >.05, η^2^ = 0.003 ), executive functions (F(2, 115) = 0.034, *p* >.05, η^2^ = 0.001), verbal memory (F(2, 118) = 0.186, *p* >.05, η^2^ = 0.028), verbal fluency (F(2, 110) = 0.747, *p* >.05, η^2^ = 0.013), language (F(2, 118) = 0.358, *p* >.05, η^2^ = 0.006) were insignificant, as well as results remained insignificant including the covariates. However, a significant interaction effect between time and group was found on general cognitive functions (F(2, 118) = 4.187, *p* <.05, η^2^ = 0.066), suggesting that egocentric perspective group showed greater improvements compared to the control group. The interaction effect remains after involving the covariates F(2, 118) = 4.187, *p* <.05, η^2^ = 0.076.

## Discussion

To the best of our knowledge, this is the first study to evaluate the significance of allocentric and egocentric perspectives in stroke patients’ cognitive rehabilitation. The results revealed that incorporating selective visual attention and short-term visual memory training tasks based on an allocentric perspective, in addition to conventional rehabilitation, led to greater improvements in short-term visual memory compared to conventional rehabilitation alone. In contrast, adding tasks based on an egocentric perspective to conventional rehabilitation resulted in greater improvements in attention-orientation compared to adding allocentric perspective tasks. These findings encourage the formulation of hypotheses suggesting that the mechanism underlying cognitive function improvement differs when cognitive training tasks based on allocentric and egocentric perspectives are incorporated into standard rehabilitation services.

The observed results, which demonstrate the effectiveness of allocentric perspective-based tasks in enhancing short-term visual memory functions, are somewhat unexpected. Thus, these findings partially contradict previous studies suggesting that individuals tend to remember information more easily from an egocentric perspective compared to an allocentric perspective [15]. However, the tasks based on both perspectives involved systematic short-term visual memory training. This suggests that allocentric perspective-based tasks more strongly activate hippocampal function compared to standard rehabilitation procedures, thereby improving short-term visual memory [35, 36]. These results align with studies indicating that the allocentric frame of reference is more closely associated with hippocampal activity, which is linked to all types of memory processes [36, 37].

The results revealed that adding tasks based on an egocentric perspective to conventional rehabilitation resulted in greater improvements in attention-orientation compared to adding allocentric perspective tasks. These results can be explained by the features of egocentric perspective which led to all possibilities for better focus during the tasks because users did not see the real environment while doing tasks in the immersive virtual reality environment. At the end of interventions, we asked the participants about their experience and most of the participants in the egocentric perspective group indicated that they had focused on the tasks integrated into the immersive virtual reality environment and did not pay attention to the real environment. These results suggest that egocentric perspective is more beneficial for attention enhancement and concentration not only on the behaviour, but other authors suggest that it also makes an impact on the neural level compared with allocentric perspective [38]. These findings would support the notion that the egocentric perspective is associated with the dorsal stream, whose activity is linked to attentional-orienting functions [39]. Consequently, tasks based on this perspective were effective in enhancing attentional-orienting abilities.

The outcomes of this experiment exposed *a near transfer effect* on spatial abilities after selective visual attention and short-term visual memory training based on egocentric and allocentric perspectives compared to conventional rehabilitation alone. These results confirm the previous research [40] that revealed near transfer effect after cognitive rehabilitation because of the overlapping functions. During both interventions used in this study, participants completed training which was based on visual stimuli that caused not only improvement in trained functions but also overlapping functions such as spatial abilities. Furthermore, during the two-week conventional rehabilitation period, participants engaged in additional cognitive activities five days per week. These activities involved tasks based on visual stimuli, which may have contributed to improvements in spatial abilities.

Additionally, tasks based on egocentric perspective caused a near transfer effect on general cognitive functions. Attention functions are considered the basis of all other cognitive functions; therefore, the results of near transfer effect after training based on an egocentric perspective can be explained by linking near transfer effect to improved attention functions, which also led to the improvement of other cognitive functions [41–43]. However, it is important to consider these results in light of tasks features such as non-immersive and immersive virtual reality environment. These findings resonate with the outcomes of prior research suggesting that tasks integrated in immersive virtual reality have a greater effect on attention and alertness in comparison with tasks integrated in non-immersive virtual reality through an increased neuroplasticity process [44, 45].

This study has several strengths, as well as some limitations. One notable strength is the inclusion of a wide age range of participants. While many studies focus primarily on older patients, this can limit the generalizability of their findings to the broader stroke population, especially since stroke incidence is increasingly observed in younger individuals [46]. According to neuroplasticity theory, younger patients tend to recover functions faster and more effectively [47]. To account for this, age was included as a covariate in the analysis. However, a larger sample size might reveal the true significance of age on the effectiveness of the intervention. Although this study was conducted specifically with stroke patients, we believe that the findings may also be applicable to other neurological populations with similar neurological disease aetiology. However, further research is needed to confirm this. It is also worth mentioning that the sample size is sufficient, especially considering the study design. Nonetheless, some very small effects, such as those related to other near transfer measures, may have been missed due to limited statistical power.

Future directions should consider the possibility of testing the long-term outcomes after 3 months or more. Additionally, it is important to include methods that assess improvements in everyday activities in a more ecological manner. For example, incorporating patients’ subjective impressions of their progress can be highly beneficial. One of the limitations that should be taken in mind is that was not implemented double-blind procedure and could affect the results. Without double-blind procedure the risk of performance, researcher and the participant bias can occur and this might contribute to the observed improvements. Furthermore, the observed effect could be due to the methodological factors, including the attention from the researcher, rather than true treatment effect. It is worth to mention that one of the limitations is the randomization method. In order to minimize the risk of allocation bias and ensure the more balanced group sizes we recommend to use other randomization methods such as number or group generator. Although our findings are derived from a specific group, they can be applied to develop care strategies for stroke patients under 85 years old with moderate to mild cognitive impairment and this can impact external validity of this study. Even the choice of exclusion criteria of patients over 85 years old was rationalised by the lack of older patients‘ motivation, increased comorbidity burdern and was based on the results of similar research [48] it is important to mention that this choice affects external validity of this study and generalizability. However, the relevance of these results may be confined to this particular subgroup, as the study’s methodology and participant characteristics might not adequately represent the experiences of older stroke patients or those with more severe cognitive impairments or additional complexities. Additional studies are needed to broaden the applicability of these findings to a wider range of stroke patients. Furthermore, future directions should consider further research in this field with a bigger sample size, which provides the opportunity to test the effectiveness in different socio-demographic groups or in isolation from other interventions such as conventional rehabilitation.

## Conclusion

In conclusion, the evidence suggests that combining tasks based on egocentric and allocentric perspectives, along with conventional rehabilitation, improves the targeted functions but also causes a near transfer effect. This aligns with and expands upon current research in the field. These results suggest that cognitive training tasks using egocentric and allocentric perspectives activate distinct neural mechanisms leading to cognitive improvement.

## Electronic supplementary material

Below is the link to the electronic supplementary material.

Supplementary Material 1

## Data Availability

The datasets used and analysed during the current study are available from the corresponding author on reasonable request.

## Abbreviations

ACE-III: Addenbrooke’s Cognitive Examination III
AP: Allocentric group
C: Control group
EP: Egocentric group
MCGCF: Medical College of Georgia Complex Figures
TMT: Trail Making Test

## References

[1] Jokinen H, Melkas S, Ylikoski R, Pohjasvaara T, Kaste M, Erkinjuntti T, Hietanen M. Post-stroke cognitive impairment is common even after successful clinical recovery. Eur J Neurol. 2015;22(9):1288–94. 10.1111/ene.12743 26040251 10.1111/ene.12743

[2] Nijsse B, Verberne DP, Visser-Meily JM, Marcel WM, De Kort PL, van Heugten CM. Temporal evolution and predictors of subjective cognitive complaints up to 4 years after stroke. J Rehabil Med. 2021;53(6). 10.2340/16501977-2840 10.2340/16501977-2840 PMC8814883 33948672

[3] Loetscher T, Potter KJ, Wong D, das Nair R. Cognitive rehabilitation for attention deficits following stroke. Cochrane Database Syst Rev. 2019;11(11):CD002842. 10.1002/14651858.CD002842.pub3 10.1002/14651858.CD002842.pub3 PMC6953353 31706263

[4] Gopi Y, Wilding E, Madan CR. Memory rehabilitation: restorative, specific knowledge acquisition, compensatory, and holistic approaches. Cogn Process. 2022;23(4):537–57. 10.1007/s10339-022-01099-w 35790619 10.1007/s10339-022-01099-w PMC9553770

[5] Katz DI, Dwyer B. Clinical neurorehabilitation: using principles of neurological diagnosis, prognosis, and neuroplasticity in assessment and treatment planning. Semin Neurol. 2021;41(02):111–23. 10.1055/s-0041-1725132 33663002 10.1055/s-0041-1725132

[6] Hickey A, Merriman NA, Bruen C, Mellon L, Bennett K, Williams D, et al. Psychological interventions for managing cognitive impairment after stroke. Cochrane Database Syst Rev. 2019;2019(8). 10.1002/14651858.CD013406

[7] Chen MH, Chiaravalloti ND, DeLuca J. Neurological update: cognitive rehabilitation in multiple sclerosis. J Neurol. 2021;268(12):4908–14. 10.1007/s00415-021-10618-2 34028615 10.1007/s00415-021-10618-2

[8] Barnett SM, Ceci SJ. When and where do we apply what we learn? A taxonomy for Far transfer. Psychol Bull. 2002;128(4):612. 10.1037//0033-2909.128.4.612 12081085 10.1037/0033-2909.128.4.612

[9] Pan DN, Hoid D, Wang XB, Jia Z, Li X. When expanding training from working memory to emotional working memory: not only improving explicit emotion regulation but also implicit negative control for anxious individuals. Psychol Med. 2022;52(4):675–84. 10.1017/S0033291720002275 32600499 10.1017/S0033291720002275

[10] Thorndike EL, Woodworth RS. The influence of improvement in one mental function upon the efficiency of other functions. II. The Estimation of magnitudes. Psychol Rev. 1901;8(4):384.

[11] Von Bastian CC, Oberauer K. Effects and mechanisms of working memory training: a review. Psychol Res. 2014;78:803–20. 10.1007/s00426-013-0524-6 24213250 10.1007/s00426-013-0524-6

[12] Klatzky RL. Allocentric and egocentric Spatial representations: definitions, distinctions, and interconnections. In: Freksa C, Habel C, Wender KF, editors. Spatial cognition: an interdisciplinary approach to representing and processing Spatial knowledge. Berlin, Heidelberg: Springer; 1998. pp. 1–17. 10.1007/3-540-69342-4_1

[13] Vogeley K, Fink GR. Neural correlates of the first-person-perspective. TiCS. 2003;7(1):38–42. 10.1016/S1364-6613(02)00003-7 10.1016/s1364-6613(02)00003-7 12517357

[14] Fernandez-Baizan C, Arias JL, Mendez M. Egocentric and allocentric spatial memory in young children: a comparison with young adults. Infant Child Dev. 2021;30(2):e2216.

[15] Tuena C, Mancuso V, Stramba-Badiale C, Pedroli E, Stramba-Badiale M, Riva G, Repetto C. Egocentric and allocentric spatial memory in mild cognitive impairment with real-world and virtual navigation tasks: a systematic review. JAD. 2021;79(1):95–116. 10.3233/JAD-201017 33216034 10.3233/JAD-201017 PMC7902987

[16] Baniña MC, Molad R, Solomon JM, Berman S, Soroker N, Frenkel-Toledo S, Levin MF. Exercise intensity of the upper limb can be enhanced using a virtual rehabilitation system. Disabil Rehabil Assist Technol. 2022;17(1):100–6. 10.1080/17483107.2020.1765421 32421460 10.1080/17483107.2020.1765421

[17] Van Muijden J, Band GP, Hommel B. Online games training aging brains: limited transfer to cognitive control functions. Front Hum Neurosci. 2012;6:221. 22912609 10.3389/fnhum.2012.00221 PMC3421963

[18] McCarthy L, Rubinsztein J, Lowry E, Flanagan E, Menon V, Vearncombe S, et al. Cut-off scores for mild and moderate dementia on the addenbrooke’s cognitive examination-III and the Mini-Addenbrooke’s cognitive examination compared with the mini-mental state examination. BJPsych Bull. 2024;48(1):12–8. 10.1192/bjb.2023.27 37272617 10.1192/bjb.2023.27 PMC10801363

[19] Ingram F, Soukup VM, Ingram PT. The medical college of Georgia complex figures: reliability and preliminary normative data using an intentional learning paradigm in older adults. Neuropsychiatry Neuropsychol Behav Neurol. 1997;10(2):144–6. 9150516

[20] Reitan RM. The relation of the trail making test to organic brain damage. J Consult Psychol. 1955;19(5):393–4. 10.1037/h0044509 13263471 10.1037/h0044509

[21] Hsieh S, Schubert S, Hoon C, Mioshi E, Hodges JR. Validation of the addenbrooke’s cognitive examination III in frontotemporal dementia and alzheimer’s disease. Dement Geriatr Cogn Disord. 2013;36(3–4):242–50. 10.1159/000351671 23949210 10.1159/000351671

[22] Sánchez-Cubillo I, Periáñez JA, Adrover-Roig D, Rodríguez-Sánchez JM, Ríos-Lago M, Tirapu J, et al. Construct validity of the trail making test: role of task-switching, working memory, inhibition/interference control, and visuomotor abilities. JINS. 2009;15(3):438–50. 10.1017/S1355617709090626 19402930 10.1017/S1355617709090626

[23] Janavičiūtė J, Paulauskas A, Šinkariova L, Blažauskas T, Kiudys E, Janonis A et al. Rationale, design and validity of immersive virtual reality exercises in cognitive rehabilitation. In: Lopata A, Gudonienė D, Butkienė R, editors. Information and Software Technologies: 28th International Conference, ICIST 2022, Kaunas, Lithuania, October 13–15, 2022, proceedings. Cham: Springer; 2022. pp. 160–70. 10.1007/978-3-031-16302-9_12

[24] Cardoso CD, Dias NM, Seabra AG, Fonseca RP. Program of neuropsychological stimulation of cognition in students: emphasis on executive functions-development and evidence of content validity. DN. 2017;11:88–99. 10.1590/1980-57642016dn11-010013 PMC5619219 29213498

[25] Jones MG. Creating electronic learning environments: games, flow, and the user interface. In: Proceedings of Selected Research and Development Presentations at the National Convention of the Association for Educational Communications and Technology (AECT). Washington: Association for Educational Communications and Technology; 1998. pp. 205–214.

[26] Maier M, Ballester BR, Verschure PFMJ. Principles of neurorehabilitation after stroke based on motor learning and brain plasticity mechanisms. Front Syst Neurosci. 2019;13:74. 10.3389/fnsys.2019.00074 31920570 10.3389/fnsys.2019.00074 PMC6928101

[27] Faria AL, Andrade A, Soares L, Badia SB. Benefits of virtual reality-based cognitive rehabilitation through simulated activities of daily living: a randomized controlled trial with stroke patients. J Neuroeng Rehabil. 2016;13(1):96. 10.1186/s12984-016-0204-z 27806718 10.1186/s12984-016-0204-z PMC5094135

[28] Gamito P, Oliveira J, Coelho C, Morais D, Lopes P, Pacheco J, et al. Cognitive training on stroke patients via virtual reality-based serious games. Disabil Rehabil. 2017;39(4):385–8. 25739412 10.3109/09638288.2014.934925

[29] Garrido LE, Frías-Hiciano M, Moreno-Jiménez M, Cruz GN, García-Batista ZE, Guerra-Peña K, et al. Focusing on cybersickness: pervasiveness, latent trajectories, susceptibility, and effects on the virtual reality experience. Virtual Reality. 2022;26(4):1347–71. 10.1007/s10055-022-00636-4 35250349 10.1007/s10055-022-00636-4 PMC8886867

[30] Brooke J. SUS: a quick and dirty usability scale. In: Jordan PW, Thomas B, Weerdmeester BA, McClelland AL, editors. Usability Evaluation in Industry. 1996.

[31] Kane JV, Barabas J. No harm in checking: using factual manipulation checks to assess attentiveness in experiments. AJPS. 2019;63(1):234–49. 10.1111/ajps.12396

[32] Faul F, Erdfelder E, Buchner A, Lang AG. Statistical power analyses using G* power 3.1: tests for correlation and regression analyses. Behav Res Methods. 2009;41(4):1149–60. 10.3758/BRM.41.4.1149 19897823 10.3758/BRM.41.4.1149

[33] Perugini M, Gallucci M, Costantini G. A practical primer to power analysis for simple experimental designs. Int Rev Soc Psychol. 2018;31(1):20. 10.5334/irsp.181

[34] Cohen J. A power primer. Psychol Bull. 1992;112:155–9. 10.1037/0033-2909.112.1.155 19565683 10.1037//0033-2909.112.1.155

[35] Castelhano J, Duarte I, Bernardino I, Pelle F, Francione S, Sales F, et al. Intracranial recordings in humans reveal specific hippocampal spectral and dorsal vs ventral connectivity signatures during visual, attention and memory tasks. Sci Rep. 2022;12(1):3488. 10.1038/s41598-022-07225-0 35241722 10.1038/s41598-022-07225-0 PMC8894428

[36] Moraresku S, Vlcek K. The use of egocentric and allocentric reference frames in static and dynamic conditions in humans. Physiol Res. 2020;69(5):787–801. 10.33549/physiolres.934528 32901499 10.33549/physiolres.934528 PMC8549915

[37] Donato F, Alberini CM, Amso D, Dragoi G, Dranovsky A, Newcombe NS. The ontogeny of hippocampus-dependent memories. J Neurosci. 2021;41(5):920–6. 10.1523/JNEUROSCI.1651-20.2020 33328296 10.1523/JNEUROSCI.1651-20.2020 PMC7880290

[38] Li G, Anguera JA, Javed SV, Khan MA, Wang G, Gazzaley A. Enhanced attention using head-mounted virtual reality. J Cogn Neurosci. 2020;32(8):1438–54. 10.1162/jocn_a_01560 32286132 10.1162/jocn_a_01560

[39] Borodaeva Z, Winkler S, Brade J, Klimant P, Jahn G. Spatial updating in virtual reality for reproducing object locations in vista space—boundaries, landmarks, and idiothetic cues. Front Psychol. 2023;14:1144861. 10.3389/fpsyg.2023.1144861 37425154 10.3389/fpsyg.2023.1144861 PMC10325663

[40] Boller B, Ouellet É, Belleville S. Using virtual reality to assess and promote transfer of memory training in older adults with memory complaints: a randomized controlled trial. Front Psychol. 2021;12:627242. 10.3389/fpsyg.2021.627242 33776848 10.3389/fpsyg.2021.627242 PMC7994284

[41] James W. The principles of psychology, volume I. New York: Holt; 1890.

[42] Kaskan PM, Nicholas MA, Dean AM, Murray EA. Attention to stimuli of learned versus innate biological value relies on separate neural systems. J Neurosci. 2022;42(49):9242–52. 10.1523/JNEUROSCI.0925-22.2022 36319119 10.1523/JNEUROSCI.0925-22.2022 PMC9761678

[43] Lambez B, Vakil E, Azouvi P, Vallat-Azouvi C. Working memory multicomponent model outcomes in individuals with traumatic brain injury: critical review and meta-analysis. JINS. 2024;30(9):895–911. 10.1017/S1355617724000468 39523448 10.1017/S1355617724000468

[44] Kumar R, Sharma G, Kumar L, Chandra S. Effect of immersion (2D vs. 3D) on attention through virtual reality. IJSR. 2017;6:204–7.

[45] Kumar J, Patel T, Sugandh F, Dev J, Kumar U, Adeeb M, et al. Innovative approaches and therapies to enhance neuroplasticity and promote recovery in patients with neurological disorders: a narrative review. Cureus. 2023;15(7). 10.7759/cureus.41914 10.7759/cureus.41914 PMC10425702 37588309

[46] Benjamin EJ, Muntner P, Alonso A, Bittencourt MS, Callaway CW, Carson AP, American Heart Association Council on Epidemiology and Prevention Statistics Committee and Stroke Statistics Subcommittee, et al. Heart disease and stroke statistics—2019 update: a report from the American Heart Association. Circulation. 2019;139(10):e56–528. 10.1161/CIR.0000000000000659 30700139 10.1161/CIR.0000000000000659

[47] Park J, Lee G, Lee SU, Jung SH. The impact of acute phase domain-specific cognitive function on post-stroke functional recovery. Ann Rehabil Med. 2016;40(2):214–22. 10.5535/arm.2016.40.2.214 27152270 10.5535/arm.2016.40.2.214 PMC4855114

[48] Tan M, Li H, Wang X. Analysis of the current status of rehabilitation motivation and its influencing factors in older adults with stroke: a cross-sectional study. Front Aging Neurosci. 2023;15:1186681. 37181623 10.3389/fnagi.2023.1186681 PMC10174289

