# Supplementary material for: Randomised control trial of virtual reality in cognitive rehabilitation: effectiveness and near-transfer effect for stroke patients
Source: BMC Psychol. 2025 Jul 18;13:805. doi: 10.1186/s40359-025-03135-8 (PMC12275414; doi:10.1186/s40359-025-03135-8)
Supplement: Supplementary file 1 — Supplementary Material 1 [file 40359_2025_3135_MOESM1_ESM.docx]

**Table S1.** Results of effectiveness on short-term memory and attention improvement within groups

| Group | Functions | N | Pre-test | | Post-test | | t | df | p**^Holm’s^** | Cohen d_z_ |
| --- | --- | --- | --- | --- | --- | --- | --- | --- | --- | --- |
|  |  |  | M | SD | M | SD |  |  |  |  |
| Target function improvement | | | | | | | | | | |
| Control | Short-term visual memory | 38 | 15.17 | 8.08 | 15.64 | 8.34 | -0.444 | 37 | .66 | 0.07 |
|  | Attention-orientation | 41 | 16.12 | 2.23 | 16.63 | 1.7 | -2.16 | 40 | **.**111 | 0.34 |
|  | Selective visual attention | 41 | 67.34 | 32.84 | 62.37 | 30.39 | 1.407 | 40 | .334 | -0.22 |
| AP | MCGCF 3-min recall score | 38 | 15.67 | 7.1 | 20.79 | 8.26 | -5.156 | 37 | **.003** | 0.84 |
|  | Attention-orientation (ACE-III) | 38 | 16.68 | 1.58 | 16.37 | 2.05 | 1.183 | 37 | .244 | **-0.19** |
|  | Visual-spatial attention (TMT-A) | 38 | 54.29 | 28.72 | 47.61 | 20.89 | 2.895 | 37 | **.012** | **-0.43** |
| EP | MCGCF 3-min recall score | 35 | 16.27 | 10.54 | 18.57 | 9.39 | -2.246 | 34 | **.031** | 0.38 |
|  | Attention-orientation (ACE-III) | 42 | 15.88 | 2.27 | 16.9 | 1.72 | -4.27 | 41 | **.004** | **0.65** |
|  | Visual-spatial attention (TMT-A) | **42** | 58.14 | 35.53 | 49.05 | 26.79 | 3.077 | 41 | **.008** | **-0.5** |

MCGCF = Medical College of Georgia Complex Figures Forms A and B; TMT = Trail Making Test Forms A and B; ACE-III = Addenbrooke's Cognitive Examination III; nVR = non-immersive VR group; iVR = immersive VR group.

**Table S2.** Results of effectiveness on short-term memory and attention improvement within groups

| Group | Functions | N | Pre-test |  | Post-test |  | t | df | p **^Holm’s^** | Cohen d_z_ |
| --- | --- | --- | --- | --- | --- | --- | --- | --- | --- | --- |
|  |  |  | M | SD | M | SD |  |  |  |  |
| Control | Visuospatial abilities (MCGCF Copy score) | 38 | 33.43 | 3.25 | 33.18 | 3.9 | 0.401 | 37 | 1 | -0.07 |
|  | Task switching (TMT-B) | 40 | 168.88 | 79.69 | 153.03 | 67.56 | 1.898 | 39 | .52 | -0.3 |
|  | Executive functions (TMT B/A) | 40 | 2.71 | 1.21 | 2.61 | 1.05 | 0.476 | 39 | 1 | -0.07 |
|  | ACE-III verbal memory | 41 | 18.68 | 5.12 | 17.76 | 5.25 | 1.830 | 40 | .525 | -0.28 |
|  | ACE-III verbal fluency | 41 | 7.9 | 2.74 | 8.32 | 2.96 | -1.371 | 40 | 1 | 0.22 |
|  | ACE-III language | 41 | 23.02 | 3.44 | 23.71 | 2.46 | -1.904 | 40 | .576 | 0.3 |
|  | ACE-III Spatial abilities | 41 | 13.66 | 2.14 | 13.71 | 2.35 | -0.208 | 40 | .836 | 0.03 |
|  | ACE-III General cognitive functioning | 41 | 79.39 | 11.32 | 80.12 | 11.03 | -0.973 | 40 | 1 | 0.15 |
| AP | Visuospatial abilities (MCGCF Copy score) | 38 | 34.68 | 2.07 | 34.62 | 1.87 | 0.158 | 37 | .875 | -0.02 |
|  | Task switching (TMT-B) | 38 | 148.61 | 73.06 | 126.82 | 60.87 | 2.585 | 37 | .098 | -0.42 |
|  | Executive functions (TMT B/A) | 38 | 2.98 | 1.35 | 2.75 | 0.88 | 0.909 | 37 | 1 | -0.15 |
|  | ACE-III verbal memory | 38 | 19.76 | 4.11 | 19.63 | 4.40 | 0.225 | 37 | 1 | -0.04 |
|  | ACE-III verbal fluency | 38 | 8.58 | 3.01 | 9.26 | 2.77 | -1.729 | 37 | .368 | 0.28 |
|  | ACE-III language | 38 | 23.76 | 2.58 | 24.63 | 1.99 | -2.809 | 37 | .064 | 0.46 |
|  | ACE-III Spatial abilities | 38 | 14.08 | 1.65 | 14.92 | 1.42 | -3.424 | 37 | **.018** | 0.55 |
|  | ACE-III General cognitive functioning | 38 | 82.87 | 9.18 | 84.82 | 10.01 | -2.212 | 37 | .198 | 0.36 |
| EP | Visuospatial abilities (MCGCF Copy score) | 35 | 32.47 | 4.93 | 32.91 | 4.27 | -0.932 | 34 | .716 | 0.16 |
|  | Task switching (TMT-B) | 40 | 140.40 | 79.86 | 119.63 | 66.87 | 2.807 | 39 | **.04** | -0.44 |
|  | Executive functions (TMT B/A) | 40 | 2.85 | 1.46 | 2.67 | 1.03 | 1.253 | 39 | .654 | -0.2 |
|  | ACE-III verbal memory | 42 | 19.07 | 4.04 | 19.57 | 4 | -0.876 | 41 | .386 | 0.14 |
|  | ACE-III verbal fluency | 42 | 8.38 | 3.32 | 9.31 | 3.35 | -2.819 | 41 | **.042** | 0.44 |
|  | ACE-III language | 42 | 23.17 | 2.92 | 24.24 | 1.90 | -3.402 | 41 | **.014** | 0.52 |
|  | ACE-III Spatial abilities | 42 | 14.21 | 2.28 | 14.71 | 1.60 | -3.532 | 41 | **.008** | 0.54 |
|  | ACE-III General cognitive functioning | 42 | 80.71 | 10.57 | 84.74 | 9.43 | -4.771 | 41 | **.000** | 0.74 |

MCGCF = Medical College of Georgia Complex Figures Forms A and B; TMT = Trail Making Test Forms A and B; ACE-III = Addenbrooke's Cognitive Examination III; nVR = non-immersive VR group; iVR = immersive VR group.
